# The potential role of scavengers in spreading African swine fever among wild boar

**DOI:** 10.1038/s41598-019-47623-5

**Published:** 2019-08-07

**Authors:** Carolina Probst, Jörn Gethmann, Susanne Amler, Anja Globig, Bent Knoll, Franz J. Conraths

**Affiliations:** 1 Friedrich-Loeffler-Institut, Federal Research Institute for Animal Health, Institute of Epidemiology, Greifswald-Insel Riems, Germany; 2 Universitäts- und Hansestadt Greifswald, Greifswald, Germany

**Keywords:** Behavioural ecology, Ecological epidemiology

## Abstract

Understanding the transmission patterns of African swine fever (ASF) among wild boar (*Sus scrofa*) is an issue of major interest, especially in the wake of the current ASF epidemic. Given the high stability of ASF-virus, there is concern about scavengers spreading infectious carcass material in the environment. Here, we describe scavenging activities on 32 wild boar carcasses in their natural habitat in Germany. Using digital cameras, we detected 22 vertebrates at the study sites, thereof two mammal and three bird species scavenging. The most frequently detected species was the raccoon dog *Nyctereutes procyonoides* (44% of all visits). Raccoon dogs, red foxes (*Vulpes vulpes*), and buzzards (*Buteo buteo*) scavenged in the warm and the cold season, while ravens (*Corvus corax*) and white-tailed eagles (*Haliaeetus albicilla*) scavenged only in the cold season. In summer, however, insects removed most of the carcass biomass. Although most of the material was consumed on the spot, foxes, raccoon dogs and ravens left the study sites in rare cases with a small piece of meat in their mouths or beaks. We conclude that scavengers represent a minor risk factor for spreading ASF, but may contribute to reducing local virus persistence by metabolizing infected carcasses.

## Introduction

African swine fever (ASF) is a viral infection of pigs and a major threat to animal health and trade. Since 2014, over 12,000 ASF cases in wild boar (*Sus scrofa*) have been registered in the Animal Disease Notification System of the European Union. In affected regions, a substantial number of wild boar die from infection, thus becoming available to invertebrate decomposers, vertebrate scavengers and susceptible conspecifics. The ASF virus (ASFV) is extremely stable in the environment and can persist at 4 °C for more than a year in blood^1^ and several weeks in pork products^2–4^. Carcasses of infected wild boar may thus be a source of infection for susceptible conspecifics until they are completely decomposed^5^. Although wild boar carcass persistence time and ASFV persistence in the soil underneath infected carcasses are still two main sources of uncertainty on ASF epidemiology, fast localization and removal of carcasses is considered as one of the most important disease control measure in affected regions. However, only a small percentage of carcasses (<10%) is usually found^6^.

Some authors consider scavenging activities as a risk factor for pathogen transmission for Tuberculosis and ASF^7^, especially since canids^8,9^ and vultures^10,11^ have been observed dispersing small pieces of carcasses. This has raised concern about the role of scavengers in ASF epidemiology. It has been proposed that reducing their populations in affected regions may be needed for effective disease control. However, this debate has less taken place in the scientific literature than in the public media.

This report is based on a study that was performed on non-protected carcasses to analyze the behaviour of wild boar towards their dead conspecifics^12^. Here, we describe temporal patterns of wild boar carcass persistence and of vertebrates visiting and feeding on them. The aim is to provide a scientific basis to decide whether scavengers need to be considered as potential mechanical vectors for ASFV within wild boar populations.

The general role of scavenging in terrestrial ecosystems has been highlighted in numerous studies and reviews, especially for the African savanna^13^, and for northern temperate regions^14,15^. Species-specific scavenging has mainly been described for the white-tailed eagle, one of the largest European raptors^16–18^, and vultures^19,20^, which do not occur in Germany. It has been shown that birds are more efficient in locating carcasses than terrestrial mammals, probably because they have the advantage of a panoramic view, in contrast to the restricted depth of view of mammals, which is caused in particular by the vegetation^21,22^. We therefore hypothesize that the first animals that detect wild boar carcasses are significantly more frequently birds (>50%) than mammals (Hypothesis 1). Accordingly, we hypothesize that the carcass detection time will be shorter for birds compared to mammals, in the cold season compared to the warm season, and in forest clearings compared to closed forests (Hypothesis 2). In Europe, carrion studies have been conducted mainly with small carcasses^23,24^ e.g. birds^25^, rats^26^ and raccoons^27^, but also with deer^28–32^, bison^33^ and domestic pigs, which are often used as proxies for human bodies in forensic research. However, most forensic studies focus on the entomology around carcasses^34,35^ and are performed by protecting them from scavengers in cages^36,37^. The decomposition process is influenced by a complex interplay of factors including temperature^38,39^, carcass size^40,41^, and integrity of the body surface^42^. We thus hypothesize that in our study carcass persistence times will depend on the body weight, carcass type, site visibility, and exposure season (Hypothesis 3). Scavenging activities represent another important factor affecting carcass persistence^43,44^. Red foxes and pine martens tend to increase scavenging at lower temperatures, whereas raccoon dogs visit carcasses more often on warm days^45^. We hypothesize that, despite species-specific differences, the total number of scavenger visits are higher in winter than in summer (Hypothesis 4).

## Results

### Animal species

**Figure 1:**
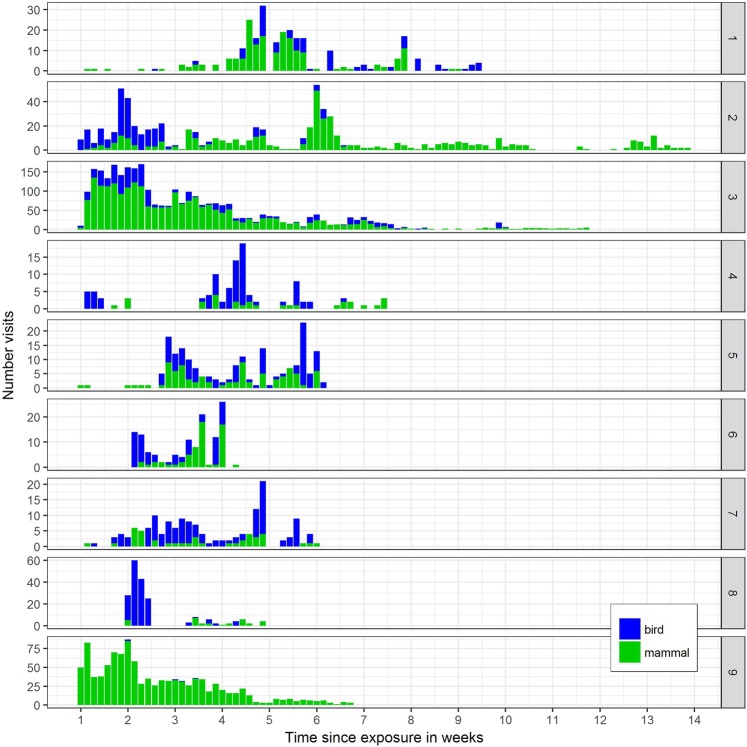
Number of visits. Number of mammal and bird visits to the wild boar carcasses per week and study site in the first 14 weeks after exposure. The number of visits to each of the eight, nine and six carcasses consecutively exposed on sites 2, 3 and 9, are aggregated.

All carcasses were subject to depredation by scavengers or decomposition by insects. Both mammals and birds were present at all nine sites (Fig. 1). Piglets 6 /7 and 8 were not tied securely enough, so they were dragged away by raccoon dogs. Carcass 1 sank unintentionally under water for three months.

The 13 cameras yielded a total of 122,160 images. The nine selected cameras yielded a total of 67,967 images that could be evaluated (i.e. at least one identifiable animal was displayed), thereof 76% (n = 51,718) in summer/autumn and 24% (n = 16,249) in winter/spring. According to the previously defined time interval of eight minutes between two visits, the images that displayed at least one identifiable animal (n = 67,967) were assigned to a total of 6,153 visits.

Altogether, 22 vertebrate species were identified, including 13 mammals and 9 birds. Two mammal scavengers were detected, i.e. the red fox (*Vulpes vulpes*) and the raccoon dog (*Nyctereutes procyonoides*); six potential mammal scavengers, i.e. wild boar, raccoon (*Procyon lotor*), marten (*Martes sp.*), polecat (*Mustela putorius*), water vole (*Arvicola terrestris*), domestic dog (*Canis familiaris*); five other mammals, i.e. European badger (*Meles meles*), red deer (*Cervus elaphus*), roe deer (*Capreolus capreolus*), red squirrel (*Sciurus vulgaris*) and horse (*Equus ferus caballus*); four scavenging birds, i.e. common raven (*Corvus corax*), common buzzard (*Buteo buteo*), white-tailed eagle (*Haliaeetus albicilla*), hooded crow (*Corvus cornix*) and the following passerines, regarded as potential scavenging birds: common blackbird (*Turdus merula*), European robin (*Erithacus rubecula*), great tit (*Parus major*), wood nuthatch (*Sitta europaea*) and common starling (*Sturnus vulgaris*). Among invertebrates, we identified the purple emperor (*Apatura iris*).

Of the images that could be evaluated, 44,470 (65%) displayed mammal scavengers, 11,203 (17%) potential mammal scavengers, 208 (0.3%) other mammals, 11,227 (17%) scavenging birds and 859 (1%) potential scavenging birds (Table 1).

**Table 1:** Number of visits to the wild boar carcasses and number of images that displayed at least one identifiable animal.

Taxonomic group/usual food source of visiting species	Species	Number of images	Number of visits	Number of visits in the first 14 weeks
Scavenging birds	common buzzard	4,652	712	637
	raven	5,786	618	595
	white-tailed eagle	789	174	157
Potential scavenging birds	northern goshawk	6	2	2
	red kite	390	73	73
	small passerines	463	97	97
Birds total		**12,086**	**1,676**	**1,561**
Mammal scavengers	fox	7,586	1,246	1,038
	raccoon dog	36,884	2,577	2,575
Potential mammal scavengers	domestic dog	18	4	4
	marten	123	48	48
	mouse	15	3	3
	raccoon	13	11	11
	wild boar	11,034	548	548
Other mammals	badger	12	4	4
	deer	193	34	34
	squirrel	3	2	2
Mammals total		**55,881**	**4,477**	**4,267**
Total		**67,967**	**6,153**	**5,828**

Small passerines include blackbird (*Turdus merula*), robin (*Erithacus rubecula*), great tit (*Parus major*), nuthatch (*Sitta europaea*), and starling (*Sturnus vulgaris*).

Of the 55,881 images of mammals, 66% (n = 36,884) displayed raccoon dogs, 20% (n = 11,034) wild boar and 14% (n = 7,586) foxes. Of the 12,086 images of birds, 48% (n = 5,786) displayed ravens, 39% (n = 4,652) buzzards, 7% (n = 859) other birds and 7% (n = 789) white-tailed eagles (Table 1).

During day and night, the study sites were meeting points of interspecies encounters. A total of 243 images displayed two species simultaneously, thereof 165 images of different birds (139 buzzard and raven; 23 raven and eagle; 3 buzzard and hawk); 67 images of different mammals (46 red fox and raccoon dog; 9 red fox and marten; 4 raccoon dog and polecat; 3 raccoon dog and marten; 3 raccoon dog and wild boar; 2 red fox and raccoon) and 11 images of a mammal and a bird (6 wild boar and a buzzard; 5 red fox and raven) (Supplementary Fig. S1).

Two mammals (foxes and raccoon dogs) and three birds (ravens, buzzards and eagles), were clearly observed scavenging on carcasses of different decomposition stages. Foxes and raccoon dogs were observed scavenging at all sites in both, the warm and the cold season. Buzzards and ravens were observed scavenging at all sites, but ravens only in the cold season. White-tailed eagles were observed only in the cold season on sites 1–3. Raccoons, different species of *Mustelidae*, water voles and several species of passerine birds were also observed for short intervals directly on the carcasses. It was not clear, however, if they had eaten carcass material.

**Figure 2:**
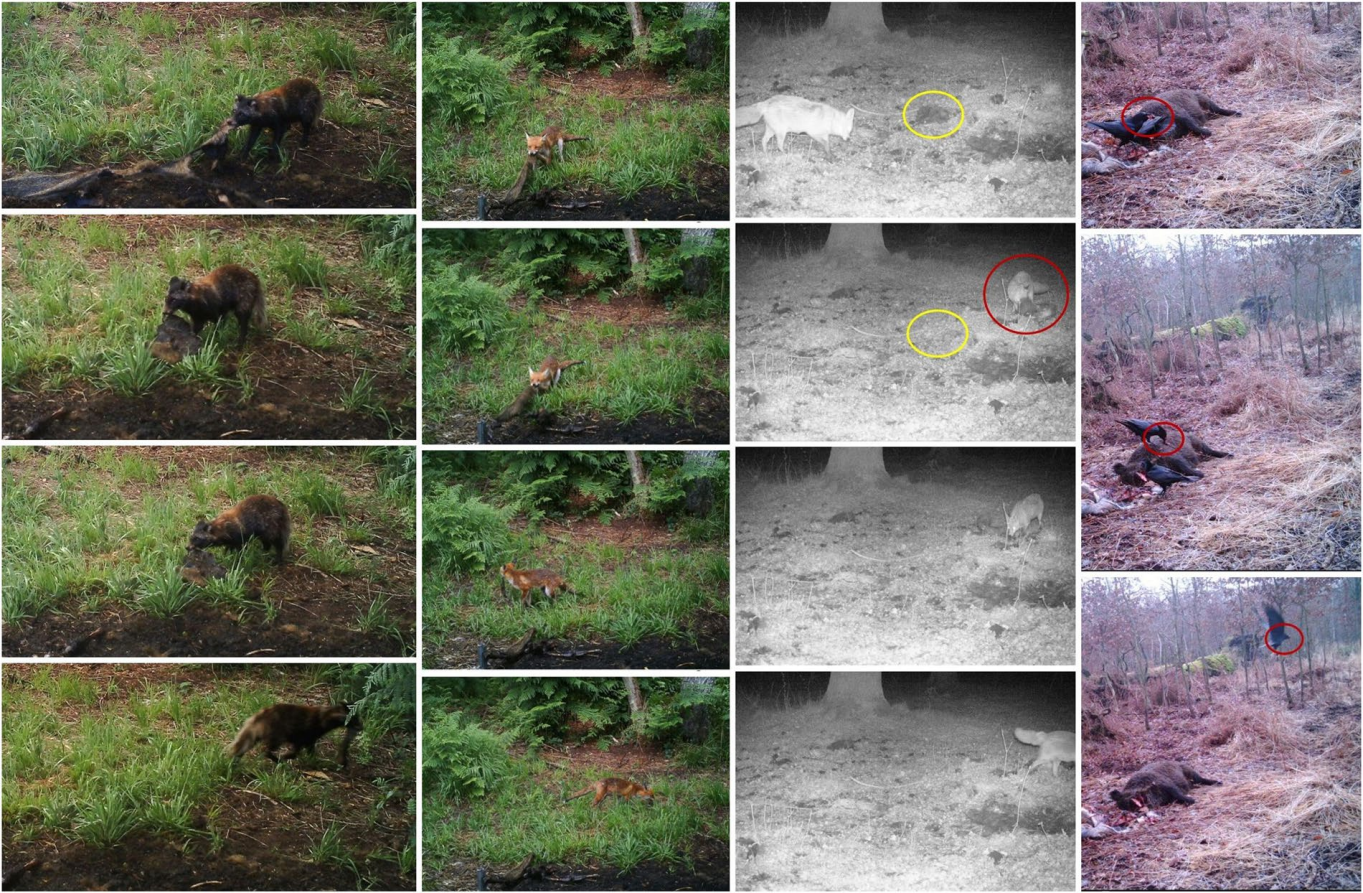
Scavengers dispersing pieces of carcasses. Top to bottom, left to right. Raccoon dog pulling at a carcass, tearing off a piece of skin and walking away with it; Fox ripping off a piece of skin of the same carcass one day later; Fox picking up a piece of carcass from the ground (yellow circle marks the location of the piece) and carrying it away; Raven leaving the visual field of the camera with a small piece of meat in the beak.

Raccoon dogs and foxes showed interest in the carcasses from day 1 after exposure until the stage when they had completely decomposed (Supplementary Fig. S2). Both species were observed vigorously tearing at carcasses and pulling intestines out of them (Supplementary Fig. S3). In advanced stages of decomposition, they were also observed ripping soft parts and skins into pieces and carrying them away (Fig. 2). Supplementary Video S6 captures foxes scavenging and pulling on carcass 12 from day 25 after exposure. Two times, ravens left the visual field of the camera with a small piece of meat in their beaks (Fig. 2). Especially during summer, raccoon dogs were observed scavenging together with their offspring in groups of up to eight individuals (Supplementary Fig. S2). Foxes scavenged individually or in pairs (Supplementary Fig. S2), raccoons were observed solitary or in groups of up to three animals (Supplementary Fig. S4). Buzzards fed and fought in pairs and ravens assembled in feeding groups of up to 14 animals (Supplementary Fig. S2).

### Hypothesis 1: The first animals that detect wild boar carcasses are significantly more frequently birds (>50%) than mammals

In 18 cases (62%, 95% Confidence Interval CI: 45–100%), the animals that first detected the carcass were birds, namely buzzards (14), ravens (2), nuthatch (1) and starling (1). In 11 cases (38%), mammals detected the carcass, namely raccoon dogs (6), wild boar (3) and foxes (2). The difference between birds and mammals was not statistically significant (95% CI: 0.45–1.00; p = 0.133, one-tailed test).

However, when the data were separately stratified by season and site visibility, in winter/spring or in forest clearings, it was mostly birds that first detected the carcasses (birds 87% vs. mammals 13%), while in summer/autumn and in closed forests, it was mostly mammals (mammals 64% vs. birds 36%; p = 0.008 for both season and site visibility). Multivariable analysis also revealed that in forest clearings and winter/spring, the chance that the carcass-detecting animal was a bird was higher (Table 2).

**Table 2:** Comparison of the full (negative-binomial including all variables) and the final model (negative-binomial after stepwise variable selection using AIC).

Risk Factors	Carcass detection by a bird				Carcass persistence time			
	Full model		Final reduced model		Full model		Final reduced model	
	OR	*p*	OR	*p*	HR	*p*	HR	*p*
Intercept	0.041		0.212					
**Season: Summer/autumn (ref)**								
Winter/spring	15.257	0.042	10.288	0.028	0.040	<0.001	0.039	<0.001
**Site visibility: Closed canopy (ref)**								
Forest clearing	11.675	0.034	10.288	0.028	0.913	0.858	NA	
**Carcass type: Open (ref)**								
Whole	0.538	0.634	NA		0.944	0.913	NA	
**Carcass body weight: ≤20 kg (ref)**								
>20–60 kg	7.001	0.241	NA		0.470	0.167	0.471	0.150
>60 kg	13.488	0.186	NA		0.294	0.064	0.285	0.040
**Model information criteria**								
AIC	34.030		30.324		119.340		115.415	
qAIC	35.156		33.688		NA		NA	
Nagelkerke’s Pseudo R^2^	0.590		0.526		0.611		0.610	
Log-Likelihood	−11.015 (df = 6)		−12.162 (df = 3)		−54.671 (df = 5)		−54.708 (df = 3)	
Δ Log-Likelihood	1.147				0.038			
Likelihood Ratio Test	0.5137				0.963			

Multivariable analysis of influencing factors on the first animal species detecting a wild boar carcass and carcass persistence time. OR, Odds Ratio; HR, Hazard Ratio; P, p-value; NA, not available.

### Hypothesis 2: Carcass detection time is shorter for birds than for mammals

The detection time ranged between 0 and 22 days in summer/autumn (median 1.5) and 0 and 16 days in winter/spring (median 6; p = 0.844). No statistically significant difference was found between mammals and birds. Mammals (n = 11) detected the carcasses between day 0 and 22 (median 4) and birds (n = 18) between day 0 and 17 (median 3; p = 0.58). Multivariable analysis with adjustment of all covariates revealed that neither body weight nor carcass type, season of exposure or site visibility had a significant influence on the carcass detection time. In closed forests, the time ranged between 0 and 22 days (median 3) and in forest clearings between 0 and 17 days (median 4; p = 0.93).

### Hypothesis 3: Persistence time of the carcass depends on the body weight, carcass type, site visibility, and exposure season

**Figure 3:**
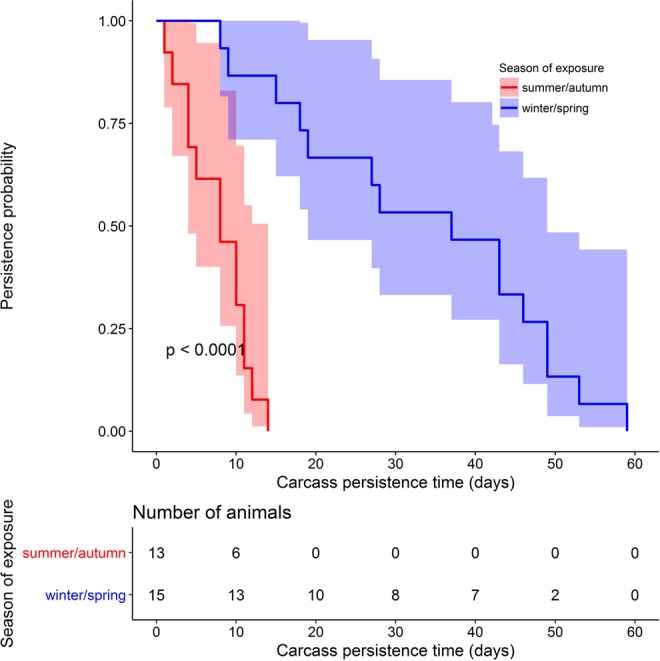
Wild boar carcass (n = 28) persistence probability in the environment with corresponding 95% confidence intervals.

Depending on the body weight and exposure season, it took between 4 days (young female wild boar exposed in summer) and 12 weeks (adult male boar exposed in autumn) until skeletonization was complete, i.e. only bones and desiccated skin were left (Table 3). For statistical analysis, carcass 1 was excluded as it had drowned in water and its decay was not comparable to that of the other carcasses. Median persistence time varied between 8 days in summer/autumn and 37 days in winter/spring (p < 0.001; Fig. 3). Univariable analyses showed no significant association with respect to body weight, carcass type and site visibility. Multivariable analyses with inclusion of exposure season, body weight, carcass type and site visibility revealed that the carcass persistence time was significantly longer in winter/spring than in summer/ autumn (adjusted Hazard Ratio HR = 0.04, 95% CI: 0.007–0.218, p < 0.001). It became also evident that heavier carcasses persisted longer than lighter ones. Cox regression with stepwise backward variable selection showed that season and body weight were the only remaining factors that explained the differences in carcass persistence time (Table 2).

**Table 3:** Wild boar carcasses exposed to scavengers.

Carcass number	Site	Visibility of the study site	Exposure season	Gender	Body weight [kg]	Carcass type	Carcass persistence time [weeks]
1	1	forest clearing	autumn	male	100	adult whole	12
2	2	closed forest	winter	male	60	adult whole	7
3	3	forest clearing	winter	male	80	adult whole	6
4,5	4	forest clearing	winter	piglet	14	piglet	6
6,7	5	forest clearing	winter	piglet	20	piglet	6
8	6	forest clearing	winter	female	30	adult eviscerated	7
9	7	forest clearing	winter	female	40	adult eviscerated	6
10	8	forest clearing	winter	piglet	15	piglet	2
11	3	forest clearing	winter	piglet	20	piglet	2
12	2	closed forest	spring	piglet	20	piglet	3
13	3	forest clearing	spring	female	35	adult whole	6
14	3	forest clearing	spring	male	80	adult whole	8
15	2	closed forest	spring	male	70	adult eviscerated	6
16	2	closed forest	spring	female	28	adult eviscerated	3
17	3	forest clearing	spring	female	26	adult eviscerated	3
18	9	closed forest	spring	male	35	adult whole	2
19	2	closed forest	summer	male	30	adult whole	2
20	9	closed forest	summer	male	62	adult eviscerated	2
21	3	forest clearing	summer	female	40	adult whole	0.5
22	2	closed forest	summer	female	50	adult eviscerated	2
23	3	forest clearing	summer	female	25	adult eviscerated	1
24	3	forest clearing	summer	male	80	adult whole	2
25	2	closed forest	summer	male	17	piglet	1
26	9	closed forest	summer	male	8	piglet	0.5
27,28	9	closed forest	summer	female	16	piglet	0.5
29	9	closed forest	summer	male	80	adult whole	1
30	2	closed forest	autumn	male	47	adult eviscerated	1
31	3	forest clearing	autumn	female	60	adult whole	1.5
32	9	closed forest	autumn	female	25	adult whole	1

### Hypothesis 4: The number of visits to the carcass depends on the visiting species, carcass type, exposure season, site visibility and time since exposure

**Figure 4:**
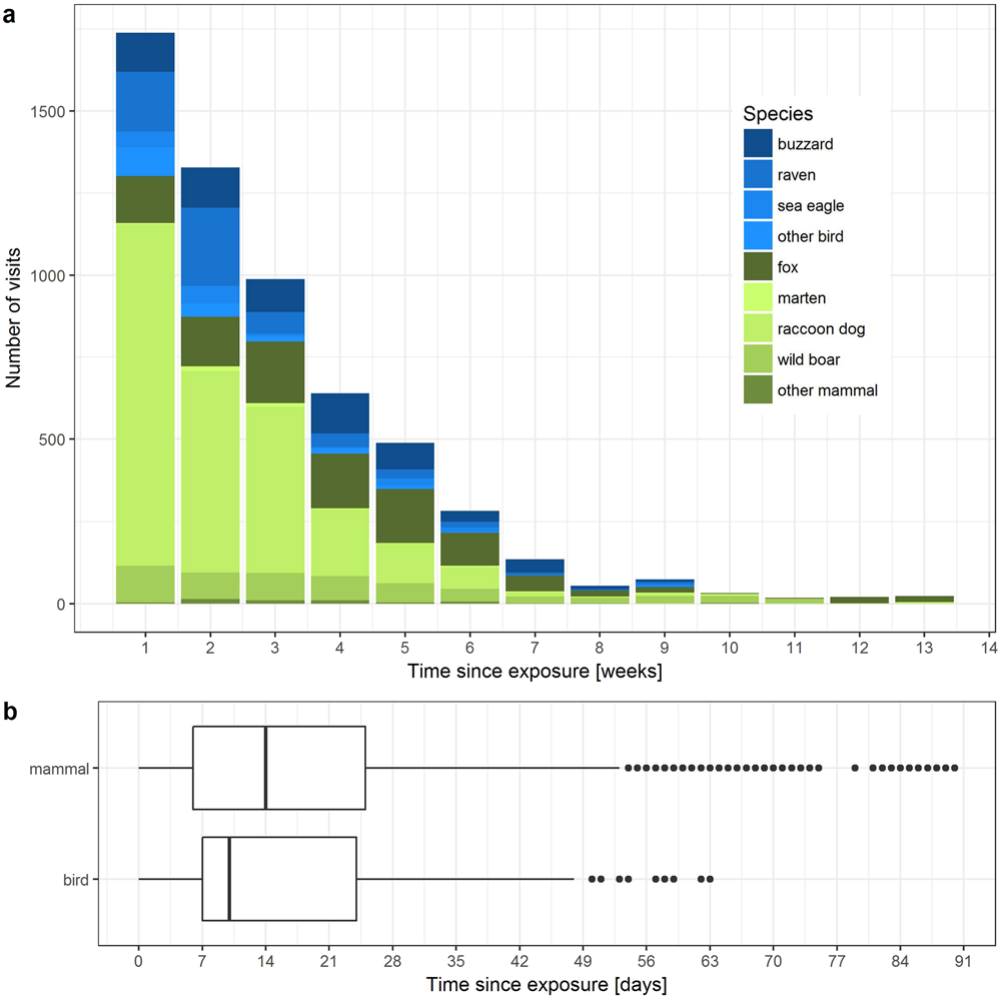
Total number of visits to the wild boar carcasses per week and species in the first 14 weeks after exposure (**a**) with corresponding boxplots (**b**).

To enhance comparability of the data, we censored all visits that took place after week 14 (98 days) after exposure. Within this period, a total of 5,828 visits were counted (Table 1). Most visits (n = 2,914) took place in the first two weeks (Fig. 4a). Half of the mammal visits took place in the first 14 days, half of the bird visits in the first 9 days (Fig. 4b).

Univariable analysis showed that mammals visited the carcasses significantly more often (n = 4,267; 73%) than birds (n = 1,561; 27%; p < 0.001). The most frequent visitors were mammal scavengers (3,613 visits; 62% of all visits), namely raccoon dogs and foxes (44% and 18% of all visits respectively), and scavenging birds (1,389 visits; 24% of all visits), namely buzzards and ravens (11% and 10% of all visits; p < 0.001; Fig. 4a). Four carcasses (20, 23, 26 and 32) were visited exclusively by mammals.

Carcasses exposed in summer were visited more often (2,354 visits; 40% of all visits) than those exposed in winter (1,656; 28%), spring (948; 16%) or autumn (870; 15%; p < 0.001). During the warm season (i.e. summer/autumn), mammals visited the carcasses more often than birds (3,104 visits; 96% vs. 120; 4%). By contrast, in winter/spring, birds visited the carcasses more often than mammals (1,441 visits; 55% vs. 1,163; 45%; p < 0.001).

Both site visibility and carcass type had also a significant influence on the number of visits: Carcasses exposed in forest clearings were visited more often than carcasses in closed forests (4,044 visits; 69% vs. 1,784; 31%; p < 0.001). Both mammals and birds were present more often in forest clearings (2,735; 64% of all mammal visits and 1,309; 84% of all bird visits) than in closed forests (1,532; 36% and 252; 16%).

Carcass 24 (adult whole male, forest clearing) was the most frequently visited (1,419 visits; 24% of all visits), whereas carcass 22 (adult eviscerated female, closed canopy) was the least frequently visited (9 visits). Whole adults were visited significantly more often (3,819; 66%) than eviscerated adults (933; 16%) and piglets (1,076; 19%; p < 0.001). The heavier the carcass, the more frequently it was visited (≤20 kg 1,076 visits, 18% of all visits; 21–60 kg 1,623 visits, 28%; >60 kg 3,129 visits, 54%; p < 0.001). Supplementary Movies illustrate an exemplary decomposition process in summer (S6, carcass 31) and fox scavenging (S7, carcass 12).

After adjusting for the effects of all other covariates, significant effects on the number of visits were found for (i) the taxonomic group (the chance to be visited by a mammal was about 1.7 times higher than to be visited by a bird), (ii) carcass type (the chance for a whole adult to be visited was 1.8 times higher than for an eviscerated adult), (iii) time since exposure (the chance for a visit was highest in the first two weeks), and (iv) site visibility (the chance for a visit in a forest clearing was 1.6 times higher than in a closed forest) (Table 4). Random effects (individual carcass number and site number) had no significant influence on the number of visits (Table 4).

**Table 4:** Final regression model (negative binomial) regarding factors that influence the number of visits to wild boar carcasses.

Risk Factors	*B*	SE	OR (exp*(B))*	95% CI for OR	*p*
**Fixed effects**					
Intercept	3.310	0.345	27.396	13.922–53.912	<0.001
**Taxonomic group: Bird (ref)**					
Mammals	0.505	0.251	1.656	1.014–2.707	0.044
**Site visibility: Closed canopy (ref)**					
Forest Clearing	0.448	0.227	1.566	1.003–2.444	0.048
**Carcass type: Adult eviscerated (ref)**					
Adult whole	0.566	0.271	1.762	1.035–2.998	0.037
Piglet	0.162	0.284	1.175	0.673–2.052	0.570
**Exposure season: Summer/autumn (ref)**					
Winter/spring	−0.298	0.244	0.743	0.460–1.198	0.223
**Time after exposure: 1–2 weeks (ref)**					
3–4 weeks	−0.416	0.243	0.660	0.410–1.062	0.087
5–6 weeks	−0.582	0.293	0.559	0.315–0.992	0.047
7–8 weeks	−1.310	0.396	0.270	0.124–0.587	<0.001
9–10 weeks	−1.343	0.493	0.261	0.099–0.686	0.00
11–12 weeks	−1.315	0.834	0.269	0.052–1.378	0.115
13–14 weeks	−0.906	1.160	0.404	0.042–3.925	0.435
**Random effects**					
σ^2^_int:carcass:site_^a^	8.055e-12				
σ^2^_int:site_^b^	7.669e-13				
**Model information criteria**					
Shape parameter θ	0.8296374				
Log Likelihood	−604.082				
AIC	1238.2				

Ref, reference; B, unstandardized regression coefficient; SE, Standard Error; OR, Odds Ratio; exp(B), exponentiated regression coefficient; CI, Confidence Interval; AIC, Akaike Information Criterion. *a* = *variance for the random intercept for carcasses nested within sites; b* = *variance for the random site-specific intercept variance*.

## Discussion

In our study, all wild carnivores known to be endemically present were observed at least once, including the raccoon dog and the red fox, which is the second largest carnivore in Germany after the wolf, *Canis lupus*, which is not a common resident in the study area. Raccoon dogs and red foxes are flexible omnivores and opportunistic scavengers with a wide-ranging diet^46,47^. We observed them scavenging in both the warm and cold season, when insects and microbes are less active, which is in line with previous studies^48,49^. Mammals mostly scavenged at night and birds during the day. This was expected, since the observed mammals are mainly nocturnal and the birds diurnal.

We found that birds were slightly faster in detecting carcasses than mammals in forest clearings and in the cold season. Birds have a number of adaptations that give them an advantage over mammals when it comes to carcass discovery, including a panoramic view, a keen sense of vision and social information transfer^19^. Ravens are usually the first species to arrive at large carcasses^50,51^ and share knowledge of feeding opportunities^52^. However, when exposing the carcasses in the field, we could not avoid leaving acoustic, olfactory and visual cues that could have made carcass detection more efficient to particular species. In addition, we might have unintentionally attracted specific scavenger species to the study sites by our peculiar behaviour. Since buzzards and ravens are communal feeders, their presence may indicate the presence of a carcass to mammal scavengers in the vicinity by their flying activities and vocalizations^21^. In our study, the largest groups that assembled at the carcasses were formed by ravens, with up to 14 individuals observed simultaneously. Previous studies found more than 20 ravens near a carcass at the same time^53^. Other studies also indicate that crows are responsible for a considerable proportion of carrion removal^26^.

In our study, the carcass detection time ranged between 0 and 22 days. We found no significant relationship between the detection time and season or the location of the carcass, namely site visibility. This is not in line with previous studies, where carcasses placed in open areas were detected and consumed at faster rates than carcasses under closed forest canopies^33^. This discrepancy might be due to the fact that on two closed forest sites, we placed several carcasses consecutively. Scavengers may thus have become habituated to this regular feeding source equally well in forest clearings and under closed forest canopies.

Although the winter was relatively mild, in the cold season the carcasses persisted several weeks or even months longer than in the warm season. Previous studies have already shown that the persistence time of a carcass is influenced by a complex interplay between weather^38,54^, type of habitat (exposure to sun or moisture, soil type etc.)^40^, presence of scavengers^55^, body size^40,41^ and integrity of the body surface^42^. Regarding the latter, we exposed 12 whole carcasses of adult wild boar. Before scavengers gained access to the inner organs and could remove larger amounts of tissues, they had to perforate the skin. The skin of wild boar, especially of adults, is much thicker than the skin of domestic pigs and seems to be particularly difficult to open. In winter, buzzards and ravens were observed picking on the torso for hours before they were able to open the first hole in the skin. Overall, in the cold season, this process was slow and tissues were preserved for several weeks. It is therefore conceivable that the carcass of wild boar that succumbed to ASF in late autumn might persist in the environment until next spring, serving then as a new source of infection. This risk was lower if the animal had died in the warm season, as the metabolization of its biomass would be significantly accelerated by invertebrates. The fact that warmer temperatures result in higher insect activity and thus in faster carcass decomposition was expected and has previously been described^56,57^.

It became obvious that insects versus vertebrates on the one hand and birds versus mammals on the other hand exhibited seasonal variability in their presence. Mammals scavenged in both, the warm and the cold season, while ravens and eagles scavenged only in the cold season. Although buzzards, ravens and eagles are resident year-round in the study area, they were mainly (eagles exclusively) observed in winter. This finding is in line with previous studies on the foraging strategy of white-tailed eagles, which documented that foraging flight duration was lowest in spring and increased over summer and autumn towards winter^16,18^. Possibly, their preferred prey (birds, fish, and small mammals)^58^ was not available in winter, so that they fed on carrion instead.

The most frequent visitors, however, were mammal scavengers, namely raccoon dogs and foxes. Not surprisingly, the season influenced their number of visits, but in the opposite way, we had expected. We had anticipated that in the cold season, when food resources are scarce, scavengers would supplement their diet more often with carrion than in the warm season. This was not the case. One reason might be that spring and summer is breeding season, during which mammalian females need an increased energy intake to cope with lactation. Especially raccoon dogs have been observed in family groups at the study sites for longer periods, probably due to the nutritional needs of the offspring. Another reason might be that we conducted the study in a region with a high population density of ungulates and a wide variety of anthropogenic food sources. Coupled with relatively mild temperatures, this may have been responsible for the low number of visits during the cold season. However, there might also be a caveat associated with this finding, since the number of carcasses exposed in summer was relatively high (n = 10).

We could not recognize a regular pattern in the sequence of scavengers or decomposition stages. The main reason was the unpredictable presence and feeding activity of scavengers. The strong influence of scavenging on the decay rate of carcasses has been noted previously^59,60^. The effects of scavenger activity were so manifold, that our idea of determining a “standard scheme of decay” for estimating the time of death of a wild boar found dead was not practicable. Also, many different individual characteristics (gender, age; gutted, wounded or with intact body surface etc.) and environmental conditions (weather, soil composition etc.) made it impossible to draw a scheme of decay or derive general rules on the “standard” scavenging behaviour towards a wild boar carcass.

Raccoons, badgers, water voles and several insectivorous birds were also observed at the study sites (Supplementary Fig. S4). We could not distinguish if they were attracted by the carcasses or by prey insects that assembled to consume organic material at the decomposition islands. Great tits, for example, have a well‐developed sense of smell^61^ and might profit from carrion opportunistically through predation on necrophagous insects. Visiting, but non-scavenging species (deer, squirrels, etc.) seemed to have had no influence on the fate of the carcasses. We identified 22 different species on the images. It was not possible, however, to identify individual animals. It is therefore possible that individual animals visited carcasses repeatedly. However, except for sites one and two, the distance between the study sites was several kilometers. It is therefore unlikely that the same animals visited different sites.

In this study, carcasses were mainly consumed at the site of exposure, except for three piglets that were dragged away. Since we tried to prevent dispersal by tying the carcasses with a rope to a pole, we cannot rule out that dragging away small carcasses is a common scavenging practice. The study design probably affected not only the number of dispersal events, but also the detectability of carcasses. For instance, under natural conditions a carcass (at least the small ones) may have been pulled into a bush, making it possibly more difficult for other animals to find or get access to it. Foxes and raccoon dogs were observed vigorously tearing the intestines out of the abdomens of carcasses of adult animals and ripping soft parts into pieces and carrying them away. In addition, ravens left the visual field of the cameras with small pieces of meat in their beaks. Red foxes and other scavengers are known for hoarding surplus food for future consumption^62,63^. We cannot quantify the amount of carcass tissue or the number of pieces that these individuals scattered around to consume them on a safe spot or to feed their young. However, from what we could see on the images, the amount of dispersed material is probably negligible. Furthermore, we assume that bones or other carcass material might be scattered over a radius representing the home range of the animal at maximum. In Mecklenburg-Western Pomerania, the study region, the average annual home range sizes of male and female raccoon dogs and red foxes are estimated between 161 and 177 ha respectively^64^. This area is smaller than the home range of wild boar family groups (the relevant species for ASF), which has been reported to vary from 29 to 685 ha and up to 3,480 ha (after hunting)^65^. Therefore, wild boar in the incubation period would anyway be more effective in spreading the disease by daily movements than scavengers by dispersing contaminated material.

During the warm season, the risk that scavengers spread ASFV might be even lower, since most carcass material is not consumed by mammals or birds, but rapidly metabolized by microbes and insects. In summer, whole carcasses were almost completely decomposed within days by carrion flies, maggots and beetles. This allowed significant amounts of nutrients to stay at the site and enter the soil, thereby forming so-called carcass decomposition islands. The relationships between these islands, decomposition, as well as insect and scavenger activity have been well described^55^.

This study has been designed as an observational study. As all experimental studies in natural settings, the results should be interpreted with caution. The sample size was limited to 32 carcasses and they had many different characteristics. Hence, the statistical procedures for analyzing the data are intended to be exploratory.

Our results show that especially in winter, raccoon dogs, foxes, buzzards and ravens play an important role in the decomposition process of wild boar carcasses, while in summer, most of the carcass biomass is rapidly decomposed by invertebrates. Although we cannot rule out that in rare cases, scavengers might disperse small pieces of infectious material in the near surroundings of a carcass in an ASF-affected region, it seems unlikely that this could have a major impact on the spread of ASF in an affected region. Previous studies suggest that scavengers may even contribute to reducing the transmission potential by removing infected material from the environment as long as they are no competent hosts for the pathogens exposed to by scavenging^66,67^. Upon ingestion, ASFV is extremely unlikely to remain infectious after passaging through the intestinal tract of a vertebrate (Sandra Blome, personal communication). Moreover, ASFV replicates in cells of the mononuclear phagocyte system of suids^68^ and certainly not on the body surface of a scavenger. In addition, mammals and birds usually groom themselves, when their fur or feathers get dirty with blood or other body fluids. Regarding the larvae of necrophagous insects, they do not play a relevant role as mechanical vectors for ASFV, but even seem to have an inactivating effect^69^.

In conclusion, we do not think that scavengers are epidemiologically relevant risk factors or that reducing the population of scavengers in ASF-affected regions is likely to have an effect on disease control. On the contrary, scavengers are efficient in removing wild boar carcasses and may thereby contribute to reducing the risk of virus persistence in the environment.

## Methods

### Study design

Thirty-two wild boar carcasses were exposed on nine sites around the town of Greifswald, northeast Germany (54°6 N, 13°23 E). The landscape is dominated by agriculture, forestry and a low number of human settlements. Ungulate density including wild boar, red deer, fallow deer (*Dama dama*) and roe deer is high. The sites were located away from paths in five different mixed coniferous/ deciduous forests dominated by oak (*Quercus robur*), alder (*Alnus glutinosa*) and beech (*Fagus sylvatica*). The forests are surrounded by crop production fields, where mainly wheat, maize, and oil-seed rape are grown. The climate is influenced by the Baltic Sea. The cold season (December to March) usually covers periods with snow and temperatures below zero. During the study period, monthly mean temperatures varied between 7.3 °C, 6.7 °C, 0.8 °C, 3.4 °C, 4.3 °C, 7.8 °C and 13.9 °C between November 2015 and May 2016, respectively. The chosen locations were particularly inaccessible away from paths. Seven sites were located in forest clearings, and two sites underneath closed canopies, i.e. shady and windless. The understorey was sparse and relatively similar across all the study sites. Only on sites 2 and 3, the herbaceous layer was dense. These sites were mown with a scythe when necessary for a free view on the carcass.

The study was conducted from 27 October 2015 until 27 October 2016, i.e. in a total of 367 days and nights. During the whole study period, no hunting took place in the surroundings of approx. 1 km of each study site. Seven carcasses (12–18) were exposed in spring, ten (19–29) in summer, four in autumn (1 and 30–32) and eight (2–11) in winter (Table 3). Fourteen carcasses were exposed under closed canopies (sites 2 and 9) and 15 in forest clearings (sites 1 and 3–8). The carcasses of piglets 4 and 5, 6 and 7 as well as 27 and 28, were exposed on the same day and close together. They were so small that each pair was counted only as a single carcass, resulting in 29 carcasses included in the statistical analysis, thereof 11 adult males, 10 adult females and 8 piglets (≤20 kg). On six sites (1 and 4–8), we placed one carcass, and on sites 2, 3 and 9, we consecutively placed eight, nine and six carcasses, respectively. All carcasses were monitored until completely consumed by scavengers or decomposed by invertebrates and only bones and skins were left over. The carcasses were either placed on the study site immediately after they had been obtained or were cooled until they could be brought to the site of exposure. They were placed directly on the forest ground so that terrestrial animals, birds and invertebrates had unrestricted access. To avoid that the carcasses were pulled outside the visual field of the cameras, they were tied up to a pole with a rope.

Each carcass was monitored either by an infrared heat- or a motion-sensitive digital camera. We used five different game camera models, namely Seissiger Special-Cam 3 Classic (Anton Seissiger GmbH, Würzburg, Germany), Maginon WK 3 HD (Supra, Kaiserslautern, Germany), Dörr Snapshot UV555 (Dörr GmbH, Neu-Ulm, Germany), Moultry A5 and Moultry I40 (Moultrie Alabaster, USA). The cameras were on standby mode during 24 hours a day and set to take one photo every 1 to 10 s if activated. They were installed on trees in a distance of 4 to 8 meters to the carcass and a height of 1 to 2.2 m above the ground. All cameras were directed and re-arranged as necessary to keep the carcasses in view and to capture all movements and animal activities in a radius of approx. 4 to 10 m around the carcasses. Date, time, and temperature were recorded automatically on each picture. In winter 2015/2016, we installed one camera at each site. To observe not only the immediate proximity, but also the surroundings of the carcasses and to capture approaching animals from all angles, we installed two additional cameras at sites 2 and 3 in summer 2016. However, as the focus of the present study was to quantify scavenger activity on the carcass itself, we used only data from one camera at each site, so that double counts of animal visits were excluded. Every two to fourteen days, the carcasses and the cameras were inspected. During each visit, pictures were taken with a handheld digital camera (Canon Power Shot A 3400) to document the status of carcass decomposition.

The Veterinary Authority of the district of Vorpommern-Greifswald was duely informed about the study and the Department of Forestry of Greifswald as the competent authority participated in it. Since no live animals were used and the wild boar carcasses had been purchased from local hunters, no ethics approval was necessary.

### Data analysis

Depending on the characteristics and spatiotemporal conditions, we divided the study sites, carcasses and visiting animals into categories. Regarding visibility from above, study sites were divided into two categories, depending on the density of the overhead canopy of branches and foliage (closed forest; forest clearing). With respect to body weight, carcasses were assigned to three categories, i.e. piglets (≤20 kg), young (21–60 kg) and adult pigs (>60 kg). With regard to the age of the animal and its status after death, the carcasses were assigned to three ‘carcass types’ (adult whole, adult eviscerated or piglet). Stratified by taxonomic group (mammal, bird), usual food sources and whether the species was observed scavenging or not, visiting animals were assigned to five categories (scavenging bird, potential scavenging bird, mammal scavenger, potential mammal scavenger and other mammal). Dates of exposure were assigned to the respective quarter of the year, namely summer (June-August), autumn (September-November), winter (December-February) and spring (March-May). For statistical analysis, only two seasons were distinguished, namely the warm (summer/autumn) and the cold (winter/spring) season.

We counted any presence of an animal on the visual field of the cameras as a “visit”. All images made of the same species in a temporal context were assigned to a single visit: If the time interval between two images was longer than eight minutes or a different species was recorded, the image was assigned to a new visit, if not, it was assigned to the same visit. If two different species were recorded at the same time, the visit was assigned to both species. Images were classified into primary visits (first visit of a species to the carcass), first direct contacts (first physical contact of that species with the carcass) and secondary visits (all following visits, regardless of involving contacts or not). Since animals might visit the study sites just by chance, i.e. without being aware of the carcass, we regarded as “carcass detection” the first visit with physical contact with the carcass (sitting on the carcass or touching it with the snout or beak). The time (days) elapsed between carcass placement and the first physical contact with the carcass was interpreted as the “carcass detection time”. “Scavenging” events were derived from the animal species involved, the observed behaviour (physical contact with the snout or beak and a clear attitude of consumption), the duration of contact and a change in the position of the carcass. As “carcass persistence time” we defined the time that elapsed from exposure to complete skeletonization (only fur and bare bones left).

To test our hypotheses, we chose the following variables: First detection of a carcass (Hypothesis 1): taxonomic group of detecting animal (bird, mammal). Carcass detection time (Hypothesis 2): taxonomic group of detecting animal, exposure season and site visibility. Persistence time of a carcass (Hypothesis 3): carcass characteristics (body weight, carcass type), site visibility and exposure season. Number of visits (Hypothesis 4): species of visiting animal, time since exposure, exposure season (as an indicator for food availability), body weight, carcass type, and site visibility. Since several carcasses were exposed at the same study sites, individual carcasses clustered on the same sites were included as random variables.

To test Hypothesis 1 (the first animals that detect wild boar carcasses are significantly more frequently birds than mammals), we used the exact binomial test for univariable analysis. To prove the influence of covariates (exposure season, site visibility, carcass type, and body weight), we tested them individually against the frequencies of birds and mammals (univariable; Fisher´s exact test). Finally, we used a logistic regression model with stepwise backward variable selection using the Akaike Information Criterion (AIC) approach to select the variables that best describe the data.

To test if the carcass detection time was shorter for birds than for mammals (Hypothesis 2), we used the log-rank test and generated Kaplan-Meier plots for univariable testing. To assess the influence of covariates (exposure season, site visibility, carcass type, and body weight), we used a Cox regression model with stepwise backward variable selection for multivariable testing.

To test Hypothesis 3 (factors that shape the persistence time of a carcass), we used the log-rank test for univariable testing. To assess the influence of covariates, we used a Cox regression model with stepwise backward variable selection for multivariable testing.

To test Hypothesis 4 (influence of the type of visiting species, time since exposure, season, body weight, carcass type, and site visibility on the number of visits), we first analyzed the distribution of the data. Since they are similar to a Poisson distribution, we first used a Poisson model with and without random effects. This model was tested for overdispersion. The results showed a significant overdispersion (p < 0.001) for the quadratic variance function, with α = 2.15. Hence, we decided to use negative binomial models with and without random effects. Finally, we applied a stepwise backward variable selection approach. The negative-binomial regression model with the lowest AIC was chosen for further analysis.

The Wald test was used to proof if the covariates´ coefficients in multivariable models were significantly different from zero. The local significance level was set to 0.05. Statistical analyses were performed using R version 3.4.1 software (R Core Team 2017). A list of all R packages used is provided in Supplementary Table S5. The two Supplementary Videos (S6 and S7) were produced using VirtualDub (GNU General Public License).

## Supplementary information

Supplementary Figures S1, S2, S3, S4 and Table S5

Supplementary Dataset 6

Supplementary Dataset 7

## Data Availability

Supporting data will be made available through https://openagrar.bmel-forschung.de

## References

[1] Plowright W, Parker J (1967). The stability of African swine fever virus with particular reference to heat and pH inactivation. Arch Gesamte Virusforsch.

[2] Kolbasov D, Tsybanov S, Malogolovkin A, Gazaev I, Mikolaychuk SV (2011). Identification of ASF virus in pork products. Veterinaria.

[3] Mebus C (1997). Survival of several porcine viruses in different Spanish dry-cured meat products. Food Chemistry.

[4] Petrini S (2019). Survival of African swine fever virus (ASFV) in various traditional Italian dry-cured meat products. Preventive Veterinary Medicine.

[5] Chenais, E. *et al*. Epidemiological considerations on African swine fever in Europe 2014-2018. *Porcine Health Management* **5**, 10.1186/s40813-018-0109-2 (2019). 10.1186/s40813-018-0109-2 PMC6325717 30637117

[6] EFSA (2015). Scientific opinion on African swine fever. EFSA Journal.

[7] Carrasco-Garcia R, Barroso P, Perez-Olivares J, Montoro V, Vicente J (2018). Consumption of Big Game Remains by Scavengers: A Potential Risk as Regards Disease Transmission in Central Spain. Frontiers in veterinary science.

[8] Haglund William D., Reay Donald T., Swindler Daris R. (1989). Canid Scavenging/Disarticulation Sequence of Human Remains in the Pacific Northwest. Journal of Forensic Sciences.

[9] Jones, A. L. *Animal scavengers as agents of decomposition: the postmortem succession of Louisiana wildlife*, Louisiana State University, Baton Rouge, LA, USA, (2011).

[10] Schultz John J., Mitchell Alexander T. (2017). Avian Scavenging of Small-Sized Pig Carcasses in Central Florida: Utilizing GIS to Analyze Site Variables Affecting Skeletal Dispersal. Journal of Forensic Sciences.

[11] Spradley MK, Hamilton MD, Giordano A (2012). Spatial patterning of vulture scavenged human remains. Forensic Sci Int.

[12] Probst Carolina, Globig Anja, Knoll Bent, Conraths Franz J., Depner Klaus (2017). Behaviour of free ranging wild boar towards their dead fellows: potential implications for the transmission of African swine fever. Royal Society Open Science.

[13] Pereira LM, Owen-Smith N, Moleón M (2013). Facultative predation and scavenging by mammalian carnivores: seasonal, regional and intra-guild comparisons. Mammal Review.

[14] Barton PS, Cunningham SA, Lindenmayer DB, Manning AD (2013). The role of carrion in maintaining biodiversity and ecological processes in terrestrial ecosystems. Oecologia.

[15] Smith JB, Laatsch LJ, Beasley JC (2017). Spatial complexity of carcass location influences vertebrate scavenger efficiency and species composition. Scientific Reports.

[16] Nadjafzadeh M, Hofer H, Krone O (2013). The Link Between Feeding Ecology and Lead Poisoning in White-Tailed Eagles. The Journal of Wildlife Management.

[17] Nadjafzadeh Mirjam, Hofer Heribert, Krone Oliver (2015). Lead exposure and food processing in white-tailed eagles and other scavengers: an experimental approach to simulate lead uptake at shot mammalian carcasses. European Journal of Wildlife Research.

[18] Nadjafzadeh Mirjam, Hofer Heribert, Krone Oliver (2015). Sit-and-wait for large prey: foraging strategy and prey choice of White-tailed Eagles. Journal of Ornithology.

[19] Kane Adam, Jackson Andrew L., Ogada Darcy L., Monadjem Ara, McNally Luke (2014). Vultures acquire information on carcass location from scavenging eagles. Proceedings of the Royal Society B: Biological Sciences.

[20] Moreno-Opo R, Trujillano A, Margalida A (2016). Behavioral coexistence and feeding efficiency drive niche partitioning in European avian scavengers. Behavioral Ecology.

[21] Kane A, Kendall CJ (2017). Understanding how mammalian scavengers use information from avian scavengers: cue from above. Journal of Animal Ecology.

[22] Ruxton GD, Houston DC (2004). Obligate vertebrate scavengers must be large soaring fliers. Journal of theoretical biology.

[23] DeVault TL, Rhodes OE, Shivik JA (2003). Scavenging by vertebrates: behavioral, ecological, and evolutionary perspectives on an important energy transfer pathway in terrestrial ecosystems. Oikos.

[24] Santos SM, Carvalho F, Mira A (2011). How Long Do the Dead Survive on the Road? Carcass Persistence Probability and Implications for Road-Kill Monitoring Surveys. PLOS ONE.

[25] Henrich M, Tietze DT, Wink M (2017). Scavenging of small bird carrion in southwestern Germany by beetles, birds and mammals. Journal of Ornithology.

[26] Inger R, Per E, Cox DTC, Gaston KJ (2016). Key role in ecosystem functioning of scavengers reliant on a single common species. Scientific Reports.

[27] Olson ZH, Beasley JC, Rhodes OE (2016). Carcass Type Affects Local Scavenger Guilds More than Habitat Connectivity. PLOS ONE.

[28] Young A, Márquez-Grant N, Stillman R, Smith MJ, Korstjens AH (2015). An Investigation of Red Fox (*Vulpes vulpes*) and Eurasian Badger (*Meles meles*) Scavenging, Scattering, and Removal of Deer Remains: Forensic Implications and Applications. Journal of Forensic Sciences.

[29] Young A, Stillman R, Smith MJ, Korstjens AH (2014). An Experimental Study of Vertebrate Scavenging Behavior in a Northwest European Woodland Context. Journal of Forensic Sciences.

[30] Gu X (2013). Wirbeltiere an Aas – Erfahrungen aus sechs Jahren Forschung in Brandenburg. Jahrbuch Nationalpark-Jahrbuch Unteres Odertal.

[31] Gu X (2014). Carcass ecology: more than just beetles. Entomologische berichten.

[32] Krofel M (2011). Monitoring of facultative avian scavengers on large mammal carcasses in Dinaric forest of Slovenia. Acrocephalus.

[33] Selva N, Jedrzejewska B, Jedrzejewski W, Wajrak A (2003). Scavenging on European bison carcasses in Bialowieza Primeval Forest (eastern Poland). Écoscience.

[34] Amendt J, Krettek R, Zehner R (2004). Forensic entomology. Naturwissenschaften.

[35] Villet, M. H. & Amendt, J. In *Forensic Pathology Reviews* (ed Elisabeth E. Turk) 213–237 (Humana Press, 2011).

[36] Matuszewski S, Bajerlein D, Konwerski S, Szpila K (2008). An initial study of insect succession and carrion decomposition in various forest habitats of Central Europe. Forensic Science International.

[37] Anton E, Niederegger S, Beutel RG (2011). Beetles and flies collected on pig carrion in an experimental setting in Thuringia and their forensic implications. Medical and Veterinary Entomology.

[38] Carter DO, Yellowlees D, Tibbett M (2008). Temperature affects microbial decomposition of cadavers (*Rattus rattus*) in contrasting soils. Applied Soil Ecology.

[39] DeVault TL, Brisbin JIL, Rhodes JOE (2004). Factors influencing the acquisition of rodent carrion by vertebrate scavengers and decomposers. Canadian Journal of Zoology.

[40] Turner KL, Abernethy EF, Conner LM, Rhodes OE, Beasley JC (2017). Abiotic and biotic factors modulate carrion fate and vertebrate scavenging communities. Ecology.

[41] Moleón M, Sánchez-Zapata JA, Sebastián-González E, Owen-Smith N (2015). Carcass size shapes the structure and functioning of an African scavenging assemblage. Oikos.

[42] Campobasso CP, Di Vella G, Introna F (2001). Factors affecting decomposition and Diptera colonization. Forensic Science International.

[43] Jennelle CS, Samuel MD, Nolden CA, Berkley EA (2009). Deer Carcass Decomposition and Potential Scavenger Exposure to Chronic Wasting Disease. The Journal of Wildlife Management.

[44] Hager SB, Cosentino BJ, McKay KJ (2012). Scavenging affects persistence of avian carcasses resulting from window collisions in an urban landscape. Scavengers at Buildings.

[45] Selva N, Jędrzejewska B, Jędrzejewski W, Wajrak A (2005). Factors affecting carcass use by a guild of scavengers in European temperate woodland. Canadian Journal of Zoology.

[46] Wikenros, C. *The return of the wolf - Effects on prey*, *competitors and scavengers*, Swedish University of Agricultural Sciences, Uppsala (2011).

[47] Needham R, Odden M, Lundstadsveen SK, Wegge P (2014). Seasonal diets of red foxes in a boreal forest with a dense population of moose: the importance of winter scavenging. Acta Theriologica.

[48] Putman, R. *Carrion and dung: the decomposition of animal wastes*. Vol. 156 (Edward Arnold, London, 1983).

[49] Wilton ML (1986). Scavenging and its possible effects upon predation - a selective review of literature. Alces.

[50] Magoun, A. J. *Summer scavenging activity in northeastern Alaska* Thesis thesis, University of Alaska, Fairbanks, AK, USA (1976).

[51] Selva, N. *The role of scavenging in the predator community of Białowieża Primeval Forest (E Poland)*, University of Sevilla, Spain (2004).

[52] Marzluff JM, Heinrich B, Marzluff CS (1996). Raven roosts are mobile information centres. Animal Behaviour.

[53] Gu, X. & Krawczynski, R. In *Proceedings of the Conference on Environmental Pollution and Public Health*, *17th–20th May* 2012. 647–649 (Scientific Research Publishing).

[54] Carter DO, Yellowlees D, Tibbett M (2010). Moisture can be the dominant environmental parameter governing cadaver decomposition in soil. Forensic Sci Int.

[55] Carter DO, Yellowlees D, Tibbett M (2007). Cadaver decomposition in terrestrial ecosystems. Naturwissenschaften.

[56] Parsons, R. H. *The Postmortem Interval: A Systematic Study of Pig Decomposition in West Central**Montana*, University of Montana (2009).

[57] Beasley, J. C., Zach, H. O. & DeVault, T. L. In Ca*rri*on Ecol*og*y, Ev*olution*, *and Their Applications* (ed Jeffery K. Tomberlin M. Eric Benbow, and Aaron M. Tarone) Ch. 107–127, 107–127 (U.S. Government Work, CRC Press, 2015).

[58] Sulkava, S., Tornberg, R. & Koivusaari, J. *Diet of the white-tailed eagle Haliaeetus allbicilla in Finland*. Vol. 74 (1997).

[59] O’Brien RC, Forbes SL, Meyer J, Dadour IR (2007). A preliminary investigation into the scavenging activity on pig carcasses in Western Australia. Forensic Sci Med Pathol.

[60] Guinard É, Julliard R, Barbraud C (2012). Motorways and bird traffic casualties: Carcasses surveys and scavenging bias. Biological Conservation.

[61] Amo L, Jansen JJ, van Dam NM, Dicke M, Visser ME (2013). Birds exploit herbivore-induced plant volatiles to locate herbivorous prey. Ecol Lett.

[62] Sklepkovych BO, Montevecchi WA (1996). Food Availability and Food Hoarding Behaviour by Red and Arctic Foxes. Arctic.

[63] Jeselnik DL, Brisbin IL (1980). Food-caching behaviour of captive-reared red foxes. Applied Animal Ethology.

[64] Drygala F, Zoller H (2013). Spatial use and interaction of the invasive raccoon dog and the native red fox in Central Europe: competition or coexistence?. Eur J Wildl Res.

[65] Sodeikat G, Pohlmeyer K (2002). Temporary home range modifications of wild boar family groups (*Sus scrofa* L.) caused by drive hunts in Lower Saxony (Germany). Zeitschrift für Jagdwissenschaft.

[66] Peisley RK, Saunders ME, Robinson WA, Luck GW (2017). The role of avian scavengers in the breakdown of carcasses in pastoral landscapes. Emu - Austral Ornithology.

[67] Cook, W. E., Williams, E. S. & Dubay, S. A. Disappearance of bovine fetuses in northwestern Wyoming. **32**, 254–259, 10.2193/0091-7648(2004)32[254:Dobfin]2.0.Co;2 (2004).

[68] Blome S, Gabriel C, Beer M (2013). Pathogenesis of African swine fever in domestic pigs and European wild boar. Virus Research.

[69] Forth JH, Amendt J, Blome S, Depner K, Kampen H (2017). Evaluation of blowfly larvae (Diptera: Calliphoridae) as possible reservoirs and mechanical vectors of African swine fever virus. Transbound Emerg Dis.

