# Author Correction: The potential role of scavengers in spreading African swine fever among wild boar

**DOI:** 10.1038/s41598-020-65547-3

**Published:** 2020-05-20

**Authors:** Carolina Probst, Jörn Gethmann, Susanne Amler, Anja Globig, Bent Knoll, Franz J. Conraths

**Affiliations:** 1 Friedrich-Loeffler-Institut, Federal Research Institute for Animal Health, Institute of Epidemiology, Greifswald-Insel Riems, Germany; 2 Universitäts- und Hansestadt Greifswald, Greifswald, Germany

Correction to: *Scientific Reports* 10.1038/s41598-019-47623-5, published online 07 August 2019

In the original version of this Article, the file provided for Supplementary Dataset 7 was incorrect. This has been corrected in the HTML version of the Article; the PDF version was correct at time of publication.

