# Supplementary material for: The potential role of scavengers in spreading African swine fever among wild boar
Source: Sci Rep. 2019 Aug 7;9:11450. doi: 10.1038/s41598-019-47623-5 (PMC6685996; doi:10.1038/s41598-019-47623-5)
Supplement: Supplementary file 1 — Supplementary Figures S1, S2, S3, S4 and Table S5 [file 41598_2019_47623_MOESM1_ESM.docx]

**The potential role of scavengers in spreading African swine fever among wild boar**

Carolina Probst^1*^, Jörn Gethmann^1^, Susanne Amler^1^, Anja Globig^1^, Bent Knoll^2^, Franz J. Conraths^1^


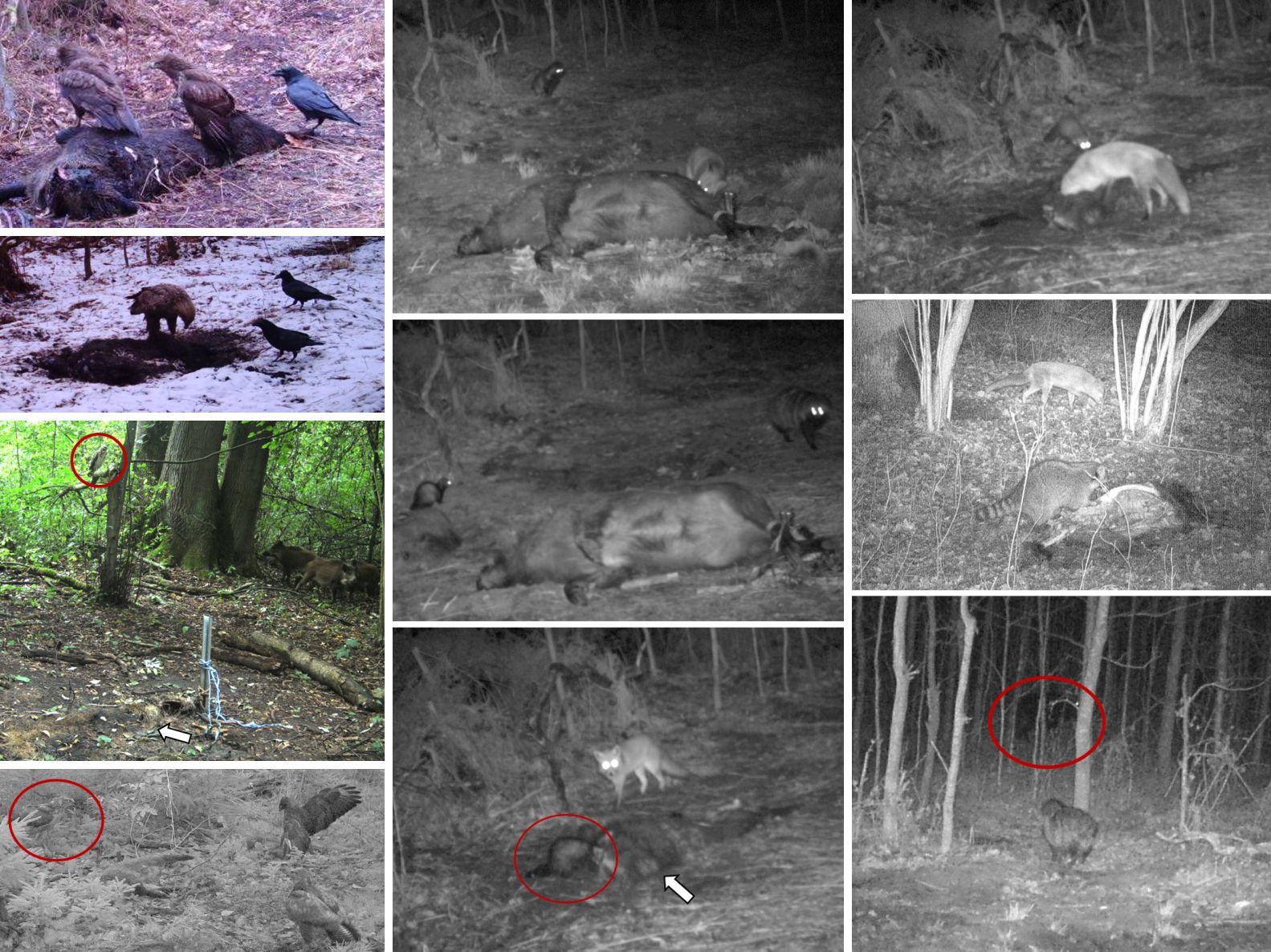


Supplementary Figure S1: Mixed species encounters. Top to bottom, left to right. Two buzzards and one raven; Two raven and one white-tailed eagle; One buzzard (red circle) and three wild boar (arrow marks the location of the carcass); Two buzzards and one hawk (red circle); Red fox scavenging, while raccoon dog is watching; Raccoon dog and polecat; Red fox arrives at carcass site while marten (red circle) is scavenging; Marten arrives at carcass site while red fox is scavenging; Red fox and raccoon; Raccoon dog stops scavenging when wild boar (red circle) arrive.


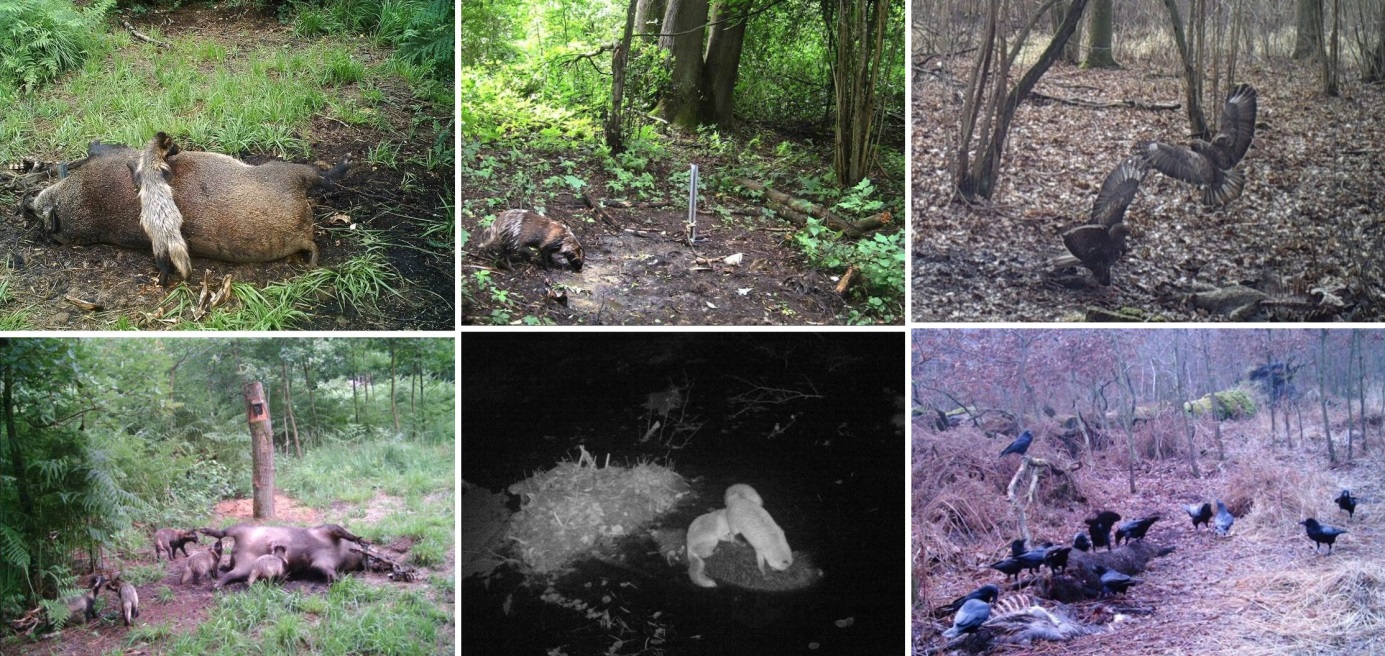


Supplementary Figure S2: Scavenging and fighting at the carcass sites. Top to bottom, left to right. Raccoon dog exploring fresh carcass 24 a few hours after exposure (note the round swollen body); Raccoon dog visiting the carcass with offspring one day later; Raccoon dog sniffing on the decomposition island of carcass 20 (note the lack of vegetation as an effect of the body fluids); Two foxes scavenging on carcass 1; Buzzards fighting at carcass 9; Group of 14 ravens at carcass 3.


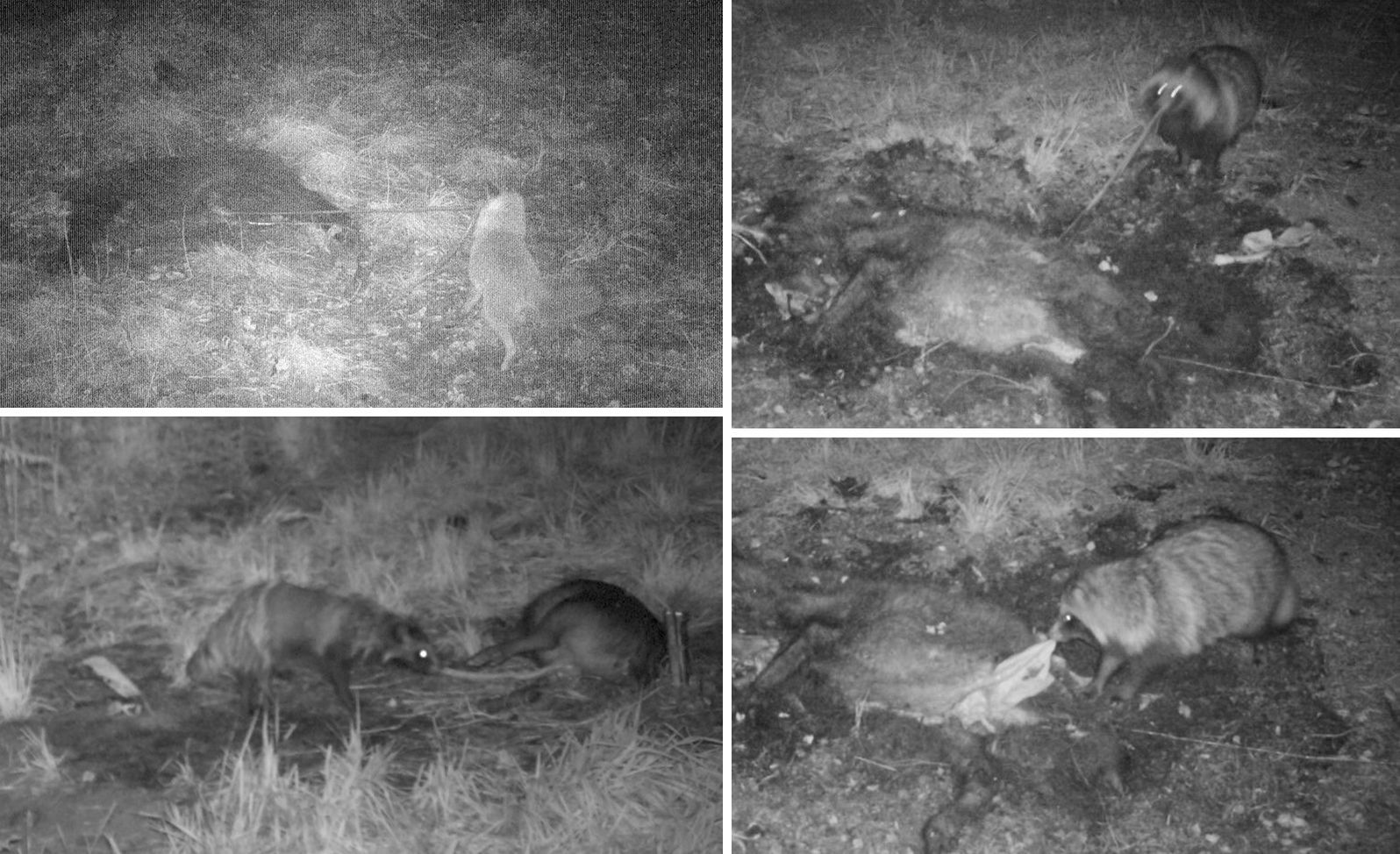


Supplementary Figure S3: Tearing out intestines. Top to bottom, left to right. Fox and raccoon dogs tearing out the intestines from the abdomen of carcasses.


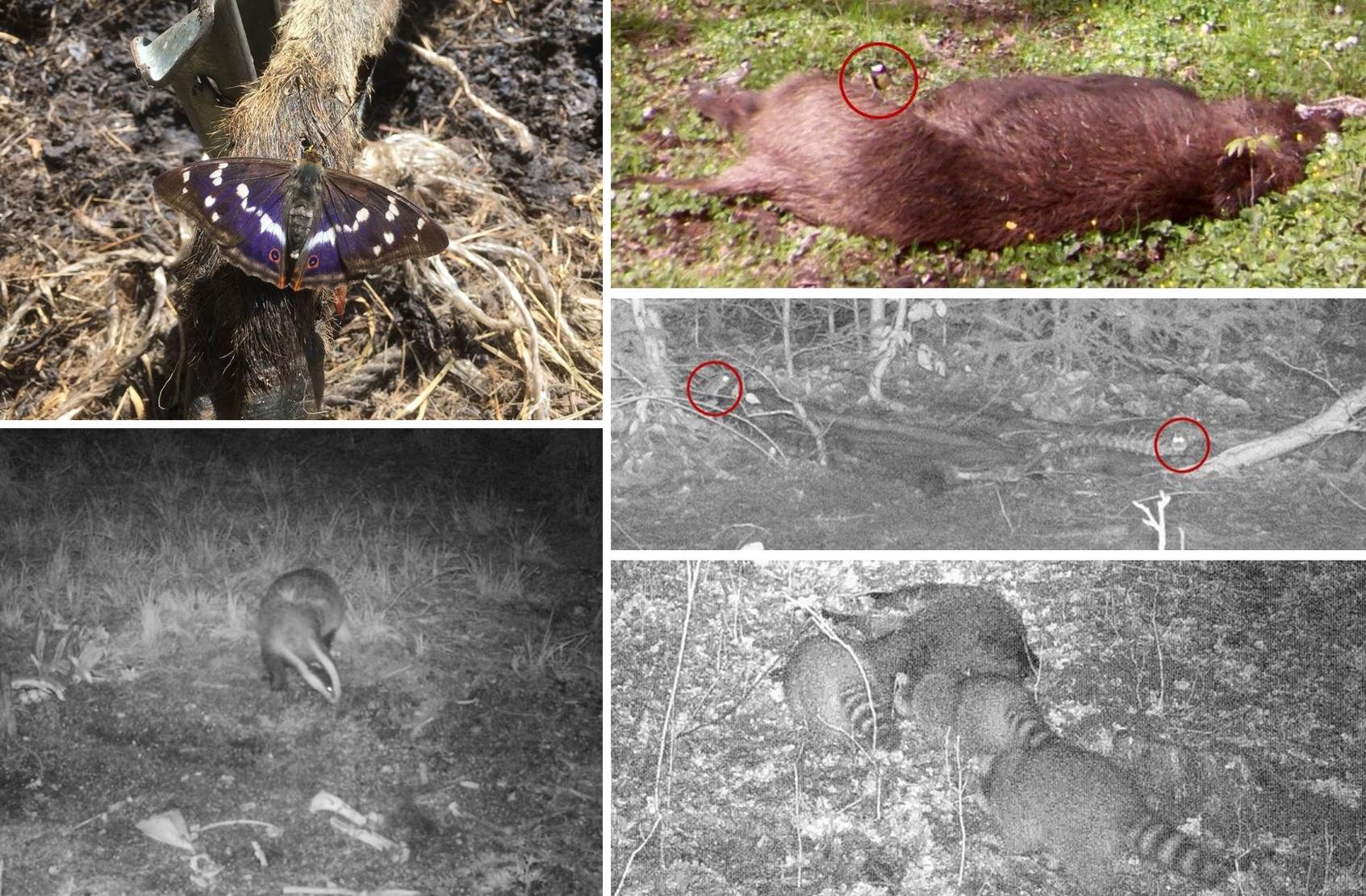


Supplementary Figure S4: Other animals approaching the carcasses. Top to bottom, left to right. Purple emperor on the leg of carcass 23; Badger on the decomposition island of carcass 24 (carcass fluids have killed the surrounding vegetation); Great tit (red circle) on carcass 15; Two water voles (red circles) on carcasses 6 and 7 (some bones and pieces of tissue are still attached to the skin of the piglets); Three raccoons at carcass 9.

**Supplementary Table S5**: List of R packages and functions used for the analyses.

| **Package** | **Version** | **Function used** | **Description** |
| --- | --- | --- | --- |
| **AER** | 1.2-6 | dispersiontest | Dispersion Test. Tests the null hypothesis of equidispersion in Poisson GLMs against the alternative of over- and/or underdispersion |
| **lme4** | 1.1-20 | fixef | Extract fixed-effects estimates |
| **lme4** | 1.1-20 | glmer | Fitting Generalized Linear Mixed-Effects Models |
| **lme4** | 1.1-20 | glmer.nb | Fitting Negative Binomial GLMMs |
| **lme4** | 1.1-20 | ranef | Extract the modes of the random effects |
| **lmtest** | 0.9-36 | lrtest | Likelihood Ratio Test of Nested Models |
| **MASS** | 7.3-51.1 | glm.nb | Fit a Negative Binomial Generalized Linear Model |
| **MASS** | 7.3-51.1 | stepAIC | Choose a model by AIC in a Stepwise Algorithm |
| **MuMIn** | 1.42.1 | model.sel | Model selection table |
| **stats** | 3.5.1 | binom.test | Exact Binomial Test |
| **stats** | 3.5.1 | fisher.test | Fisher's Exact Test for Count Data |
| **stats** | 3.5.1 | glm | Fitting Generalized Linear Models |
| **stats** | 3.5.1 | kruskal.test | Kruskal-Wallis Rank Sum Test |
| **stats** | 3.5.1 | logLik | Extract Log-Likelihood |
| **stats** | 3.5.1 | wilcox.test | Wilcoxon Rank Sum and Signed Rank Tests |
| **survival** | 2.43-3 | coxph | Fit Proportional Hazards Regression Model |
| **survival** | 2.43-3 | survdiff | Test Survival Curve Differences |
